# Epstein-Barr Virus-Positive T/NK-Cell Lymphoproliferative Diseases in Chinese Mainland

**DOI:** 10.3389/fped.2018.00289

**Published:** 2018-10-09

**Authors:** Junhong Ai, Zhengde Xie

**Affiliations:** Beijing Key Laboratory of Pediatric Respiratory Infectious Diseases, Key Laboratory of Major Diseases in Children, Ministry of Education, National Clinical Research Center for Respiratory Diseases, National Key Discipline of Pediatrics (Capital Medical University), Beijing Pediatric Research Institute, Beijing Children's Hospital, Capital Medical University, National Center for Children's Health, Beijing, China

**Keywords:** Epstein-Barr virus, T/natural killer cell, lymphoproliferative disorder, clinical feature, China

## Abstract

Epstein-Barr virus-positive T/NK-cell lymphoproliferative disorders (EBV^+^ T/NK LPD) encompass a heterogeneous group of disorders, including chronic active Epstein–Barr virus infection (CAEBV), Epstein-Barr virus-associated hemophagocytic lymphohistiocytosis (EBV-HLH), systemic EBV^+^ T-cell lymphoma of childhood and hydroa vacciniforme-like lymphoproliferative disorder (HVLPD) and so on, predominantly affecting children and young adults with high mortality. Patients with EBV^+^ T/NK LPD have overlapping clinical symptoms as well as histologic and immunophenotypic features. In this review, we summarized the clinical features of EBV^+^ T/NK LPD in Chinese patients from the published articles.

## Introduction

Epstein-Barr virus (EBV)-positive T/natural killer (NK)-cell lymphoproliferative disorder (EBV^+^ T/NK LPD) encompasses a heterogeneous group of disorders, including chronic active Epstein–Barr virus infection (CAEBV), Epstein-Barr virus-associated hemophagocytic lymphohistiocytosis (EBV-HLH), systemic EBV^+^ T-cell lymphoma of childhood and hydroa vacciniforme-like lymphoproliferative disorder (HVLPD) and so on. EBV^+^ T/NK LPD are rare, predominantly affect children and young adults, and associated with high mortality. To date, only hematopoietic stem cell transplantation (HSCT) has been shown to be promising for EBV^+^ T/NK LPD patients, including those not yet having progressed to lymphoma (1). In this review, we summarized the clinical features of EBV^+^ T/NK LPD in Chinese patients including CAEBV, EBV-HLH, systemic EBV^+^ T-cell lymphoma of childhood and HVLPD.

## Chronic active epstein–barr virus infection (CAEBV)

CAEBV has been defined as a systemic EBV-positive lymphoproliferative disease (EBV^+^ LPD) characterized by fever, lymphadenopathy, and splenomegaly developing after EBV infection in patients without known immunodeficiency. CAEBV is more common in children than in adults.

In China, there were only two retrospective studies on the clinical features of CAEBV systematically, one is about pediatric patients and the other is about adult cases (2, 3). In total, eighty one CAEBV patients were reported, including 53 children with a mean age of 6.3 years (ranging from 6 months to 15 years) and 28 adults with a median age of 45 years (ranging from 20 to 81 years). The male to female ratios were 2.12 and 0.75 in children and adults, respectively.

The clinical features and complications of CAEBV in Chinese patients are summarized in Table S1. The most frequent signs and symptoms of CAEBV were fever, splenomegaly, hepatomegaly and lymphadenopathy. Life-threatening complications mainly included hemophagocytic syndrome, hepatic failure, and interstitial pneumonia. The peripheral blood count depletions are common in CAEBV patients. In China, one study about pediatric CAEBV patients showed that there was an imbalance in lymphocyte subsets and disturbance in cellular immunity. The number of lymphocytes, NK cell, B cell, total T cell, CD4^+^ T cell, and CD8^+^ T cell in CAEBV were lower than that in acute EBV infection (4). In adult onset CAEBV patients, the B cell, NK cell, CD4^+^ T cell and CD8^+^ T cell counts were also decreased (3).

The clinical characteristics of pediatric CAEBV cases were different from that of adult patients in China. The prevalence of hemophagocytic syndrome was lower in pediatric patients than in adult patients. Unlike pediatric cases reported, the manifestations of cardiovascular diseases in adult patients included pulmonary arterial hypertension, decreased cardiac function and aorta vasculitis. A comparison with Japanese CAEBV (5) was also made in Table S1. The incidences of lymphadenopathy and interstitial pneumonitis were comparatively higher and the prevalence of hypersensitivity to mosquito bites was comparatively lower in Chinese patients than in Japanese patients.

CAEBV can be classified into the T-cell, NK-cell and B-cell types, depending on which lymphocyte subset is mainly infected with EBV. However, in Chinese CAEBV patients, EBV infected cell types were analyzed in only small fraction of patients. In a study, in seven out of 10 CAEBV patients the virus infected cell type was detected, with six cases in T cells, and one in NK cells (6).

Although CAEBV occurred in individuals without apparent immunodeficiency, primary HLH associated immune gene mutations were detected in some CAEBV patients in China, such as heterozygous mutations in *PRF1, UNC13D*, and *STXBP2* (7). Furthermore, a pediatric patient with atypical primary immunodeficiency (PID) was reported with CAEBV as the initial symptom (8). Thus, genetic background may play a role in this disease.

There were few studies on the treatment of CAEBV in China. Retrospective studies showed that the prognosis of CAEBV is poor. Without HSCT, only 12.0% patients (5/42) experienced remission for 1 to 3 years after the onset of the disease and 26.2% (11/42) patients died 7 months to 3 years after onset because of the life-threatening complications, such as hemophagocytic syndrome, malignant lymphoma and hepatic failure and so on (2).

## Epstein-barr virus-associated hemophagocytic lymphohistiocytosis (EBV-HLH)

HLH is an immune disorder characterized by uncontrolled T lymphocyte and macrophage activation and an excessive production of inflammatory cytokines. EBV-HLH is the most frequent subtype of secondary HLH triggered by infections. Eligibility criteria for EBV-HLH were as follows: (1) meeting HLH-2004 diagnostic criteria (9), (2) high level of EBV viral load in the peripheral blood or tissues or number of cells containing EBV-encoded small RNA (EBER) in the peripheral blood or tissues.

In China, most of the studies on EBV-HLH were retrospective (10–12). There was no exact number of EBV-HLH cases in China because the overlap among different studies conducted in the same hospital. EBV-HLH was more common in pediatric patients than in adults, with the age of onset from 2 months to 78 years (12, 13). Over all, male was more likely to develop EBV-HLH than female.

Active EBV-HLH develops rapidly with a high mortality rate if reasonable and effective interventions are not undertaken. The initial therapies of EBV-HLH used in China included antiviral therapy, glucocorticosteroid, symptomatic therapy, HLH-94, and HLH-04 regimen. Antiviral therapy was also used in some EBV-HLH patients in China (10, 12, 13), but the exact benefit of antiviral therapy was not shown in these studies. The treatments of EBV-HLH in China are shown in Table 1. The response rate showed that HLH-94 and HLH-04 regimens were more effective. Without chemotherapy, the prognosis of EBV-HLH was very poor.

**Table 1:** Treatment of EBV-HLH in China.

**Treatments**	**Number of cases (*n*)**	**Gender (male/female)**	**Median age (range), y**	**Previous treatment of HLH**	**Disease status before treatment**	**Prognosis**	**Response/survival rate (%)**	**References**
Glucocorticoid	21	/	/	No	Initial diagnosed	3 years OS: (36.2 ± 14.7)%	36.2 ± 14.7	(11)
Symptomatic treatment	18	/	/	No	Initial diagnosed	3 years OS: 0	0	(11)
HLH-94	33	/	/	No	Initial diagnosed	5 CR, 12 PR, 16 NR	51.5	(12)
HLH-04	16 44	/ /	/ /	No No	Initial diagnosed Initial diagnosed	1 CR, 6 PR, 9 NR 3 years OS: (55.8 ± 7.9)%	43.8 55.8 ± 7.9	(11) (12)
DEP	22	16/6	30.5 (18–57)	HLH-94± rituximab	refractory	5 CR, 11 PR, 6 NR,	72.7	(14)
L-DEP	28	22/6	24 (7–50)	HLH-94± rituximab	refractory	9 CR, 15 PR, 4 NR and dead	85.7	(15)
Allo-HSCT	14 13	9/5 /	19 (14–55) /	HLH-94 L-DEP	10 remission, 4 unremission 9 CR,4 PR	9 alive, 5 dead 10 alive, 3 dead	64.3 76.9	(16) (15)
Haploid HSCT	30	20/10	32 (18–55)	HLH-94, salvage therapies	10 CR, 10 PR, 10 NR	19 survival, 11 dead	63.3	(17)

*/, not reported; OS, overall survival; CR, complete response; PR, partial response; NR, no response; allo-HSCT, allogeneic hematopoietic stem-cell transplantation; salvage therapies: DEP, pegaspargase-DEP, or CHOP; Response rate: CR+PR or survival*.

In refractory EBV-HLH after the therapy of HLH-94, a salvage therapy DEP regimen (including liposomal doxorubicin, etoposide, and high-dose methylprednisolone) was used and achieved better efficacy with overall response rates (complete and partial response) of 72.7% (14). However, the duration of response after DEP regimen is relatively short and there is a significant risk of gastrointestinal bleeding. A modified PEG-aspargase and DEP regimen combination therapy (L-DEP) was used in refractory EBV-HLH as the salvage therapy (15). The overall response rate of L-DEP regimen was 85.7%. It seems that L-DEP is a safe and effective salvage therapy prior to allo-HSCT (allogeneic hematopoietic stem-cell transplantation) for refractory EBV-HLH and increases the possibility of such patients receiving allo-HSCT. A prospective multicenter large-scale clinical trial that aims to validate the L-DEP regimen for refractory EBV-HLH is currently underway (ClinicalTrails.gov Identifier: NCT02631109) (15).

For refractory EBV-HLH, allo-HSCT should be used as early as possible. In China, the survival rates of allo-HSCT were 64.3 and 76.9% after the HLH-94 and L-DEP regimen, respectively (15, 16). Haploidentical HSCT was also used in Chinese adult EBV-HLH patients. The 3-year overall survival rate of haploidentical HSCT was 63.3% (17).

## Systemic EBV^+^ T-cell lymphoma of childhood

Systemic EBV^+^ T-cell lymphoma of childhood is a life-threatening illness in children and young adults, and is characterized by the clonal proliferation of EBV infected T cells with an activated cytotoxic phenotype. Its name used to be systemic EBV^+^ T-cell LPD of childhood in the 2008 World Health Organization (WHO) classification of lymphomas (18) and changed to systemic EBV^+^ T-cell lymphoma of childhood in 2016 WHO Classification of lymphoid neoplasms (19).

In China, 3 pediatric cases with systemic EBV^+^ T-cell lymphoma were reported (20, 21) (Table S2). The common clinical features of this disease were fever and hepatosplenomegaly. A special patient manifested as gastrointestinal disorders and skin lesion progressed from CAEBV (T-cell type) to systemic EBV^+^ T-cell lymphoma of childhood was reported (21).

The prognosis of systemic EBV^+^ T-cell lymphoma is poor. Among the 3 cases reported in China, 2 of them died. One patient experienced rapid progression and died within 5 months of onset (20). The other one died of intestinal hemorrhea (21).

## Hydroa vacciniforme-like lymphoproliferative disorder (HVLPD)

HVLPD is a rare type of EBV^+^ lymphoproliferative disorder of cytotoxic T-cell or NK-cell origin that mainly affect children, characterized by a vesicopapular skin eruption that clinically resemble hydroa vacciniforme (HV). The disease is reported to more frequently affect Asians and Latin Americans. Its name used to be hydroa vacciniforme-like lymphoma in 2008 World Health Organization (WHO) classification of lymphomas (18) and changed to HVLPD in 2016 WHO classification of lymphoid neoplasms (19).

In China, 31 patients with HVLPD were reported and their clinical features were summarized in Table 2 (22–31). Among them, 20 patients were children with the age ranging from 3 to 15 years old, and 11 patients were adult with the age ranging from 18 to 74 years old.

**Table 2:** Clinical features of hydroa vacciniforme-like lymphoproliferative disorder (HVLPD) reported in China.

**Number of cases**	**Age (year)**	**Gender**	**History of recurrent skin lesions (year)**	**Treatment/prognosis**	**References**
7	Ranging from 6 to 14	5M/2F	Ranging from 0.17 to 13	Patient 1: acitretin, prednisone and traditional Chinese medicine/alive with occasional skin eruptions; patient 2: prednisone and traditional Chinese medicine/alive with occasional skin eruptions; patient 3: prednisone and chemotherapy/dead of disease; patient 4: traditional Chinese medicine/alive with occasional skin eruptions; patient 5: IFN/alive with occasional skin eruption; patient 6: prednisone/alive with disease with no changes; patient 7: prednisone and IFN/alive with disease with condition improved	(22)
7	Mean 7.43 (ranging from 3 to 12)	2 M/5F	Mean 2.79 (ranging from 0.5 to 6)	Four cases were treated with IFN-α with skin eruptions improved; and 2 patients were treated with chemotherapy with condition got worse; one case lost follow up	(23)
2	8, 11	M, F	2, 3	One was treated with IFN-α with remarkable clinical improvement at the 6-month follow up; and another was treated with Tibetan medicine with 6-month follow-up, part of her skin eruptions had become smaller and regressed	(24)
1	6	F	3	Treated with cyclosporine and CHOP with condition stable for 6 months	(25)
1	14	F	6	Treated with acyclovir and IFN-α with marked improvement	(26)
2	15, 14	M, M	3, 6	One received glucocorticoid treatment with an improvement of the edema and the cutaneous lesion, and another received glucocorticoid treatment with no sign of recurrence or extracutaneous involvement during 36 months follow up	(27)
6	Ranging from 20 to 58	4M/2F	Ranging from 0.25 to 7	Patient 1: lost to follow up/dead of disease; patient 2: prednisone, IFN, rapamycin, and etoposide/dead of disease; patient 3: prednisone and IFN/dead of disease; patient 4: prednisone/alive with partial remission; patient 5: chemotherapy, IFN, cyclophosphamide/dead of disease; patient 6: IFN/alive with occasional skin eruptions	(22)
2	19, 18	F,F	6, 10	One was treated with chemotherapy and died of disease; Another was treated with chemotherapy and alive with disease	(28)
1	74	M	Over a year	Treated with topical corticosteroids and with good improvement	(29)
1	19	M	2	Treated with Chinese homeopathic medicine, ketotifen, IFN-α, Levofloxacin, sirolimus, and prednisone, died 3 month later	(30)
1	48	M	0.58	Treated with hydroxychloroquine, IFN-α, and prednisone with symptoms began to regress gradually	(31)

*M, male; F, female; IFN, interferon; IFN-α, interferon-α*.

HVLPD patients often had a long history of recurrent skin lesions before systemic manifestations. In Chinese HVLPD patients, the history of recurrent skin lesions ranged from 2 months to 13 years (22). Many therapies have been applied for the treatment of HVLPD, including interferon (IFN), traditional Chinese medicine, acitretin, acyclovir, prednisone, prednisolone, and chemotherapy. IFN and glucocorticoid were used more commonly and always made an improvement of the disease (22–24, 26). Chemotherapy is uncommonly used because the temporary improvement of disease and the worsened condition of patients after the use of it (22, 23, 25). The prognosis of HVLPD was not well. In all patients reported in China, 20 cases (64.5%) had condition improved after therapy, 6 cases (19.4%) died, 3 cases (9.7%) got worse after therapy, 1 case (3.2%) had no change after therapy and 1 case (3.2%) lost follow up.

In conclusion, EBV-positive T/NK-cell lymphoproliferative disorders encompass a heterogeneous group of disorders which have a common feature with excessive lymphoid proliferation of mainly T cells and/or NK cells. They often have overlapping clinical symptoms as well as histologic and immunophenotypic features because both T and NK lymphoid cell types derive from a common precursor.

## Limitation of the mini review

There were some limitations in this mini review. First, in China EBV infected lymphocyte lineages were only characterized in small part of CAEBV patients, not characterized in EBV-HLH and HVLPD patients. It has been shown that clinical feature of CAEBV are different between T-cell type and NK-cell type. So it is difficult to compare the clinical features of Chinese CAEBV with Japanese CAEBV without considering EBV infected cell types. Second, the treatment and outcome of some CAEBV cases were not fully described in references.

## Author contributions

All authors listed have made a substantial, direct and intellectual contribution to the work, and approved it for publication.

### Conflict of interest statement

The authors declare that the research was conducted in the absence of any commercial or financial relationships that could be construed as a potential conflict of interest.

## Abbreviations

DEP: doxorubicin-etoposide-methylprednisolone
L-DEP: DEP regimen in combination with PEG-aspargase.
