# Supplementary material for: Epstein-Barr Virus-Positive T/NK-Cell Lymphoproliferative Diseases in Chinese Mainland
Source: Front Pediatr. 2018 Oct 9;6:289. doi: 10.3389/fped.2018.00289 (PMC6189562; doi:10.3389/fped.2018.00289)
Supplement: Supplementary file 1 [file Table_1.DOCX]

Table S1. Comparison of the clinical features and complications of CAEBV among Chinese pediatric and adult cases and Japanese cases

| Categories | Chinese pediatric CAEBV (n=53) (Lu et al., 2009) | Chinese adult CAEBV (n=28) (Luo et al., 2018) | Japanese CAEBV (n=82) (Kimura and Cohen, 2017) |
| --- | --- | --- | --- |
| Age at admission (range) | Mean 5.3 years (2 months-14.6 years) | median 45 years (20- 81 years) | Mean 11.3 years (9 months -53 years) |
| Male/female ratio | 2.12 | 0.75 | 1.05 |
| Fever (%) | 92.5 | 100 | 93 |
| Lymphadenopathy (%) | 69.9 | 85.7 | 40 |
| Splenomegaly (%) | 77.4 | 89.2 | 73 |
| Hepatomegaly (%) | 81.1 | 42.8 | 79 |
| Hypersensitivity to mosquito bites (%) | 5.7 | 0 | 13 |
| Hemophagocytic syndrome (%) | 24.5 | 57.1 | 24 |
| Hepatic failure (%) | 15.1 | 25 | / |
| Interstitial pneumonitis (%) | 24.5 | 25 | 5 |
| Malignant lymphoma (%) | 11.3 | / | / |
| Acute leukemia (%) | 1.9 | / | / |
| Intestinal hemorrhage | / | 7.1 | / |
| Disseminated intravascular coagulopathy (DIC) (%) | 3.8 | 3.6 | / |
| CNS disease (%) | 9.4 | 10.7 | 9 |
| Digestive tract ulcers (%) | 1.9 | 7.1 | / |
| Cardiovascular diseases |  |  |  |
| (1) Pulmonary arterial hypertension (%) | / | 14.2 | / |
| (2) Severe pulmonary arterial hypertension (%) | / | 7.1 | / |
| (3) Decreased cardiac function (%) | / | 3.6 | / |
| (4) Aorta vasculitis (%) | / | 3.6 | / |
| (5) Coronary artery aneurysms | 1.9 | / | / |
| (6) Valvular disease | 1.9 | / | / |

Note: /, not reported.

Table S2. Clinical features of systemic EBV^+^ T-cell lymphoma in China

| Patient number | Age (year) | Gender | Clinical presentation | Histopathology | Treatment | Prognosis | Reference |
| --- | --- | --- | --- | --- | --- | --- | --- |
| 1 | 14 | Male | Fever, hepatosplenomegaly, lymphadenopathy, anemia, sensitivity to insect bites | Lymph node structure was partially destroyed, neoplastic cells proliferated actively in an expanded T-zone | Chemotherapy, interferon | Alive. Currently completely healthy, 38 months from onset | (Huang et al., 2014) |
| 2 | 9 | Male | Fever, hepatosplenomegaly, lymphadenopathy, pancytopenia, then progressed to multiorgan failure | Lymph node structure was totally destroyed, follicle dendritic cell was dimin­ished or disappeared, neoplastic cells diffusedly infiltrated | Chemotherapy | Dead at 5 months from onset | (Huang et al., 2014) |
| 3 | 14 | Male | Digestive disorders, intermittent fever, skin lesion, hepatosplenomegaly, lymphadenopathy,  anemia, hypoalbuminemia, repeatedly spontaneous intestinal perforations | Diffuse heterotypic lymphoid cells infiltration; karyorrhexis and patchy necrosis | Mesalazine and prednisone, supportive treatment, emergency resection and anastomosis | Died of intestinal hemorrhea | (Xiao et al., 2016) |
